# 肺癌合并眼部转移：单中心回顾性研究并文献综述

**DOI:** 10.3779/j.issn.1009-3419.2017.05.05

**Published:** 2017-05-20

**Authors:** 燕 徐, 伊多 孙, 静 赵, 闽江 陈, 蒋德利娜, 龙芸 李, 巍 钟, 孟昭 王

**Affiliations:** 1 100730 北京，中国医学科学院，北京协和医学院，北京协和医院呼吸内科 Department of Respiratory Medicine, Peking Union Medical College Hospital, Chinese Academy of Medical Sciences and Peking Union Medical College, Beijing 100730, China; 2 100730 北京，中国医学科学院，北京协和医学院，北京协和医院风湿免疫科 Department of Rheumatology and Immunology, Peking Union Medical College Hospital, Chinese Academy of Medical Sciences and Peking Union Medical College, Beijing 100730, China

**Keywords:** 肺肿瘤, 眼部肿瘤, 肿瘤转移, Lung neoplasm, Eye neoplasms, Neoplasm metastasis

## Abstract

**背景与目的:**

眼部转移（ocular metastasis）是肺癌少见转移之一，影响患者生活质量。本研究旨在探讨肺癌合并眼转移患者的临床特征及预后。

**方法:**

回顾性分析肺癌合并眼部转移9例患者的临床资料，回顾近10年文献报道共42例患者的临床特点。

**结果:**

9例患者的中位年龄为51岁（范围：41岁-61岁），其中7例诊断为非小细胞肺癌（包括腺癌6例）；1例为小细胞肺癌；1例病理不详。眼转移部位方面，脉络膜8例，虹膜1例。文献回顾中，小细胞肺癌占21.4%（*n*=9），腺癌占47.6%（*n*=20），脉络膜是最常见的眼转移部位（66.7%, *n*=28）。肺癌合并眼转移患者，系统化疗的疾病控制率仅为28%，联合眼部局部治疗可有效控制眼部症状。

**结论:**

肺癌合并眼部转移以肺腺癌多见，脉络膜转移是肺癌眼部转移的最常见转移部位。眼部局部治疗可控制局部症状，但系统性化疗疗效差。

## Abstract (en)

**Background and objective:**

Eye is a rare site of lung cancer metastasis, and ocular metastasis is one of the largest challenges to cancer patients' quality of life (QOL). Here we present our experience on ocular metastasis of lung cancer and review relevant literature in an attempt to investigate the clinical features, treatment, and prognosis of these tumors.

**Methods:**

The records of 9 patients with ocular metastasis of lung cancer treated at our hospital were analyzed. A literature review identified 42 cases reported in the last 10 years and their medical records were retrospectively estimated.

**Results:**

The median age of our patients was 51 years (range 41-61). Diagnosis of lung cancer included non-small cell lung carcinoma (NSCLC) in 7 patients, in which adenocarcinoma (ADC) were recorded in 6 patients, small cell lung carcinoma (SCLC) in 1 patient, and unknown in 1 patient. The site of ocular metastasis included choroid (*n*=8) and iris (*n*=1). In the literature review, SCLC constituted 21.4% (*n*=9) and ADC constituted 47.6% (*n*=20). Choroid presented to be the most common site for eye metastasis (66.7%, *n*=28). As for disease control rate, systemic chemotherapy for lung cancer patients with ocular metastasis presented to be only 28%. Meanwhile, combination of systemic treatment with ocular treatment could improve patients' eye symptoms effectively.

**Conclusion:**

The most common lung cancer that metastasizes to the eye is ADC. The choroid is the most common site for ocular metastasis. Ocular treatment can improve patients' eye symptoms, while the effect of systemic chemotherapy treatment is limited.

肺癌常见的转移部位包括脑转移、骨转移、肝转移、肾上腺转移、肺内转移、胸腔转移等^[1]^。眼部转移（ocular metastasis）是肺癌少见转移之一，肺癌眼部转移发生率大约0.1%-7%^[2-4]^。恶性肿瘤葡萄膜转移是最常见的眼内转移^[5]^，其中以脉络膜转移最为常见，其次是虹膜转移、睫状体转移，此外，眼眶转移、眼睑转移、结膜转移^[6]^、视网膜转移^[7]^以及视神经转移均有报道。恶性肿瘤眼部转移的症状^[5]^包括视物模糊/视力下降、疼痛、飞蚊症、视野缺损，肿块、红眼、闪光感、复视等，亦有大约11%-23%患者是无症状的^[5, 8]^。由于肿瘤转移导致的视觉障碍，严重影响患者生活质量（quality of life, QOL），缩短患者生存期。

对于以眼部症状为首发表现的肺癌患者，眼部转移及肺部原发肿瘤诊断相对困难，诊断周期相对较长。同时，合并眼部转移的肺癌患者，无论对于眼部转移病灶局部治疗和肺癌系统性治疗，均有极大的难度。肺癌合并眼部转移，在诊断和治疗方面，对眼科医生和肿瘤科医生都是巨大的挑战，需进一步提高对肺癌眼部转移的认识，缩短诊断时间，提高诊断率，并进一步制定针对性的局部治疗策略和系统性的抗肿瘤治疗策略。

本研究回顾我中心肺癌并合并眼部转移的患者，总结其临床特征，并回顾文献，总结文献中报道的相关病例，对肺癌合并眼转移患者临床特征进行整理和分析，以期指导后续临床。

## 1 资料及方法

### 1.1 患者资料

以“转移性眼部恶性肿瘤”及“支气管恶性肿瘤”为检索关键词，于我院病案室进行检索，1988年1月-2016年12月期间，共检索到9例肺癌合并眼部转移的患者，收集9例患者临床资料。

### 1.2 研究方法

收集患者病例资料，回顾患者临床信息，肺癌相关信息包括：性别、年龄、肺癌症状、肺癌诊断时间、病理、分期、转移部位、肺癌相关治疗、治疗疗效及转归。眼部转移相关信息：眼部转移症状、眼部转移诊断时间、眼部转移至肺癌诊断的时间，眼部受累侧、受累部位、视力、眼压、眼部检查（眼底检查、眼部超声、眼部核磁、眼底荧光造影）、病理结果、眼部治疗、治疗疗效和转归。

### 1.3 文献回顾

以“Eye Neoplasms”、“eye, eyelid, orbital, uvea, choroid, ciliary body, iris”、“metastasis”以及“lung neoplasms”为检索关键词，采取主题词与自由词相结合的方式，对Pubmed数据库2007年1月-2016年12月肺癌合并眼部转移文献进行检索，本文共检出个案报道48篇、病例总结6篇，精读全文剔除临床信息缺失的文献（个案报道12篇及病例总结5篇），最终纳入文献整理个案报道34篇、病例总结1篇，纳入本研究共计42例患者。整理个案患者的临床信息：性别、年龄、眼部转移部位、是否以眼部转移起病、肺癌病理类型、肺癌转移部位、眼部转移瘤治疗及疗效、系统性抗肿瘤治疗及疗效、以及总生存期（overall survival, OS）。

### 1.4 统计学方法

应用SPSS 17.0统计软件进行统计学分析及进行统计学绘图。计量资料应用Mean±SD表示，计数资料采用率表示。

## 2 结果

### 2.1 基本临床特征

1988年1月-2016年12月期间，共9例患者入院诊断为肺癌合并眼部转移。男性患者5例，女性患者4例，中位年龄51岁（范围：41岁-61岁）。3例患者有吸烟史。

### 2.2 眼部转移临床表现及检查（表 1）

**1:** 9例肺癌合并眼转移患者临床特征 The clinical manifestations of 9 lung cancer patients with eye metastasis

Case No.	Sex/age (yr)/smoking status	Ocular symptoms				Ocular examination				Ocular Involvement	Metastatic Sites (exept eye)	Durationbetween the onset of symptoms and admission (months)	Histology	Ocular treatment/Response	SystemicTreatment/Response
		Blurred vision	Elevated intraocular pressure	Visual field defect	Floater	Fundus photograph	Ultrasound	MRI	FFA						
1	M/50/Y	Y	Y			Y	Y			BE/Choroid	Adrenal, bone, liver	3 m	ADC	Surg/SD	CTx-NS/SD
2	F/60/N	Y			Y	Y	Y	Y	Y	RE/Choroid	Brain	6 m	ADC	None	None
3	F/47/N	Y			y	Y	Y	Y		RE/Choroid	Bone	2 m	ADC	None	Chemo(GP)/NA
4	M/51/Y	Y	Y	Y		Y		Y	Y	RE/Choroid	Bone	4 m	ADC (EGFR 19del)	Intravitreal Bev/PD; RT/PR	Gefitinib/PR
5	F/57/N	Y				Y	Y	Y	Y	RE/Choroid	Lung	3 m	ADC(EGFR-WT, ALK (-))	None	Chemo(AP)/NA
6	F/47/N	Y	Y			Y	Y			RE/Choroid	Adrenal, liver, lung	4 m	ADC	Surg/NA	None
7	M/54/N	Y	Y	Y		Y		Y		RE; Choroid	No	3 m	NA	None	None
8	M/41/N	Y							Y	LE/Choroid	Bone, pleura	2.5 m	NSCLC	None	None
9	M/61/Y	Y				Y	Y			LE/Iris	Liver, pleura	-5 m	SCLC	None	Chemo(Topotecan)/NA
ADC: adenocarcinoma; AP: pemetrexed-cisplatin; BE: bilateral eyes; Bev: bevacizumab; Chemo: chemotherapy; CTx-NS: chemotherapy (not specified); EGFR: epidermal growth factor receptor; F: female; GP: gemcitabine+cisplatin; LE: left eye; M: male; N: no; NSCLC: non-small cell lung carcinoma; NA: data not available/not presented; RE: right eye; PR: partial response; RT: radiation therapy; SCLC: small cell lung carcinoma; SD: Stable disease; Surg: surgery; WT: wild type; Y: yes.															

9例患者中，8例患者出现视力下降，2例患者出现飞蚊症，2例患者视野缺损，4例患者眼压升高、伴有头痛，诊断为继发性青光眼。针对此9例患者眼部检查进行分析，有8例患者行眼底检查，其中7例患者可见视网膜白色或黄色隆起，其中5例伴有视网膜浅脱离，1例患者见虹膜菜花样新生物；有6例患者行眼部超声，5例诊断为脉络膜占位性病变，1例患者诊断左眼虹膜占位性病变；有4例患者完善眼底荧光血管造影（fundus fluorescein angiography, FFA），诊断脉络膜转移癌；有4例患者行眼部核磁检查，结果提示眼球后壁梭形或弧形短T1短T2信号向球内突出，诊断为脉络膜占位。9例患者中，8例患者诊断脉络膜转移，1例患者诊为虹膜转移；1例患者双眼受累，6例右眼受累，2例左眼受累。

### 2.3 肺癌临床特征（表 1）

9例患者中，7例患者诊断为非小细胞肺癌（non-small cell lung carcinoma, NSCLC）（其中6例患者为腺癌），1例患者诊断为小细胞肺癌，1例患者病理结果不详。7例患者以眼部症状为首发表现，眼部症状出现至肺癌的诊断中位时间为3个月（2.5个月-6个月）；此外，NO.1患者为手术后5年，后眼部症状为首发复发表现；NO.9患者为SCLC一线化疗后复发过程中发现眼部转移。诊断眼部转移时，1例患者只有眼部转移，无其他部位转移；其他患者均有不同部位的全身转移。对于眼部局部治疗，2例患者采取手术治疗，1例患者采用局部贝伐珠单抗注射及放疗。全身治疗方面，4例患者行系统性化疗，其中1例患者疗效为稳定（stable disease, SD），其余患者疗效不详；1例检测到表皮生长因子受体（epidermal growth factor receptor, EGFR）exon 19del突变（图 1），给予吉非替尼治疗，全身评估疗效部分缓解（partial response, PR），眼部经过放疗及系统性治疗，高眼压症状明显缓解。

**1:**
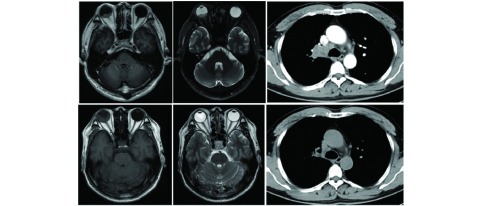
NO.4患者影像学结果：吉非替尼及眼部放疗治疗前后头颅核磁及胸部CT变化。基线：头颅核磁示右眼底稍短T1短T2信号（A和B）；胸部增强CT右上叶开口占位，纵膈淋巴结增大（C）。治疗后（吉非替尼及眼部局部放疗）：患者眼部疼痛缓解，右眼底病变的大小和信号均有变化（D和E）；右肺病变较前缩小（F）。 Imaging findings of patient NO. 4: changes of brain magnetic resonance (MRI) imaging and chest computed tomography (CT) before and after gefitinib treatment and local (ocular) treatment. Initial presentation: Brain MRI images at the initial presentation revealed a mass in the choroid of the right eye (A and B). Chest computed tomography scan at the initial presentation revealed a mass in the upper lobe of the right lung with mediastinal lymphadenopathy (C). After treatment (gefitinib treatment and radiation therapy): Brain MRI images after treatment revealed the choroidal metastasis changed in shape and signal (D and E), with the patient's eye aching improving. Chest computed tomography scan after treatment revealed the shrinking of the tumor in the right lung (F).

### 2.4 文献回顾

42例患者纳入文献回顾（表 2）。患者中位年龄为54岁（range 25-77）。其中女性20例（20/42, 47.6%），男性22例（22/42, 52.4%），28.6%（*n*=12）有明确吸烟史，23.8%（*n*=10）患者无吸烟史，另有47.6%（*n*=20）患者吸烟史不详。以眼部症状起病的患者占76.1%（*n*=32）。

**2:** 42例文献回顾肺癌患者合并眼转移患者临床特征 Clinical data of 42 Reviewed lung cancer cases with eye metastasis

Patient No	Age /sex /smoking status	Ocular Involvement	Tumor location	Onset with Ocular symptom	Type of lung cancer	Metastatic Sites* (exept eye)	Ocular Treatment/ Response	Systemic Treatment/response	OS (mo)
1^[9]^	68/M/N	LE	Choroid	Y	ADC	Bone	Intravitreal Bev/Progression	Chemo (AC)+Bev/PR	2.1 mo
2^[10]^	40/M/N	RE	Choroid	N	ADC	Brain	NA	NA	NA
3^[11]^	44/F/N	LE	Choroid	Y	SqCC	Neck LN; liver, bone	None/Regression	Crizotinib/PR	> 12 mo
4^[12]^	71/M/NA	RE	Ciliary body	N	NSCLC	None	NA	CTx-NS/NA	NA
5^[13]^	28/F/NA	BE	Choroid	N	SqCC	NA	NA	Chemo (NP)/SD	10 mo
6^[14]^	36/F/NA	LE	Choroid	Y	ADC	NA	Intravitreal Bev/Regression	Chemo (TC)+Bev/SD	> 9 mo
7^[15]^	52/M/NA	RE	Iris	Y	SCLC	Brain; adrenal	Intravitreal Bev/Regression	Chemo (Irinotecan)/SD	> 9 mo
8^[6]^	50/M/Y	BE	Upper eyelid; conjunctiva	Y	NSCLC-NOS	Brain	None	None	5 mo
9^[16]^	35/M/NA	LE	Choroid	Y	ADC	NA	Enucleation/Regression	Surg/PR	36 mo
10^[17]^	52/F/NA	RE	Choroid	Y	NETs	None	RT/Regression	Surg/PR	> 18 mo
11^[18]^	51/F/Y	RE	Choroid	Y	ADC	None	EBRT/Regression; Enucleation/Regression	Chemo (GC)/PD	7 mo
12^[19]^	57/M/Y	LE	Orbit	Y	SCLC	None	ERBT/Regression	CTx-NS/PD	10 mo
13^[19]^	60/M/Y	RE	Orbit	Y	SCLC	Bone	RT/Unchanged	CTx-NS /PD	6 mo
14^[20]^	42/F/Y	BE	Choroid	Y	ADC	Bone; brain; meningeal	None	Chemo (TP)/NA	NA
15^[21]^	50/M/N	LE	Choroid	Y	ADC	Adrenal; mesentery	RT/NA	CTx-NS /PD	9 mo
16^[22]^	42/F/NA	RE	Choroid	Y	LCC	Neck LN	None	Chemo (GC)+Bev/PR	NA
17^[23]^	57/F/NA	LE	Choroid	N	ADC	NA	Intravitreal Bev/Regression	Erlotinib /SD	> 4 mo
18^[24]^	61/F/NA	BE	Optic nervers	Y	ADC	Brain	RT/Regression	None/PD	32 mo
19^[25]^	46/M/Y	LE	Choroid	Y	SqCC	Adrenal; brain; bone	RT/NA	CTx-NS+RT/NA	NA
20^[26]^	55/M/Y	RE	Choroid	Y	ADC	Bone	RT/Regression	CTx-NS/PD	> 3 mo
21^[27]^	56/M/NA	BE	Choroid	Y	SCLC	Liver	None	None	NA
22^[28]^	69/M/N	LE	Optic disc	Y	Ad-sq	None	Enucleation+RT/Unchanged	None/PD	4 mo
23^[29]^	70/F/Y	RE	Intraocular cavities	Y	ADC	Liver, large bowel	None	Chemo (AP)/PD	NA
24^[30]^	69/F/NA	LE	Iris	Y	SCLC	Adrenal; brain; abdominal LN	Intravitreal Bev/Regression	Chemo (Irinotecan) /NA	8 mo
25^[31]^	59/F/NA	LE	Choroid	N	NETs	NA	RT/Regression	NA	13 mo
26^[31]^	77/F/NA	RE	Choroid	Y	NETs	Liver	RT/Regression	None	> 4 mo
27^[31]^	77/F/NA	BE	Choroid	Y	NETs	None	Enucleation/Unchanged	None/SD	22 mo
28^[32]^	34/F/NA	LE	Choroid	N	ADC	None	Intravitreal Bev/Regression	Chemo (GP) /SD	> 20 mo
29^[33]^	73/M/Y	LE	Choroid	Y	ADC	NA	Intravitreal Bev/Unchanged	Erlotinib/PD	4 mo
30^[34]^	25/M/Y	BE	Choroid	Y	ADC	Liver; bone	EBRT/Progression; ERBT/Unchanged	Chemo (DP) /PD	NA
31^[35]^	60/M/N	LE	Choroid	Y	ADC	None	None	Chemo (AP)/PR	> 11 mo
32^[35]^	42/F/N	LE	Choroid	Y	LCC	Liver	Intravitreal Bev/Regression	Chemo (TP)/PR	9 mo
33^[35]^	53/M/Y	RE	Choroid	Y	ADC	Liver, adrenal; bone	Intravitreal Bev/Progression; Intravitreal Bev +RT/regression	Chemo (AP)/PD; Chemo (DC)+Bev/PR	16 mo
34^[36]^	62/F/N	BE	Choroid	Y	ADC	LNs from neck to pelvis	None/progression; RT/Unchanged	Chemo (AC)/PD; Erlotinib/PR; Crizotinib/PR	6 mo
35^[37]^	73/F/NA	RE	Optic nerve	Y	ADC	Brain; bone	Enucleation/Regression	CTx-NS/NA	> 6 mo
36^[38]^	64/M/NA	RE	Iris	Y	NETs	Adrenal; bone; brain; abdominal LN	Intravitreal Bev/Regression	CTx+RT-NS/PD	> 6 mo
37^[39]^	43/M/NA	BE	Choroid	Y	ADC	Bone	None	Crizotinib/PR	> 48 mo
38^[40]^	58/M/NA	RE	Iris	Y	SCLC	None	RT/Regression	Chemo (EP)/PR	NA
39^[41]^	60/F/N	RE	Choroid	Y	ADC	Bone	Intravitreal Bev/Regression	Chemo (AP)→gefitinib/PR	> 5 mo
40^[41]^	49/M/N	BE	Choroid	Y	SCLC	Liver	Intravitreal Bev/Progression; EBRT/NA	Chemo (IP)/SD; Topotecan/NA	NA
41^[42]^	65/F/NA	LE	Retina	Y	SCLC	NA	Intravitreal Bev/Progression	CTx-NS/PD	NA
42^[43]^	46/M/Y	BE	Orbits; optic nerve	N	SCLC	Brain	RT/Progression	NA	NA
AC: carboplatin+pemetrexed; Ad-sq: adenosquamous carcinoma; AP: pemetrexed-cisplatin; BE: bilateral eyes; Bev: bevacizumab; CTx-NS: chemotherapy (not specified); DC: docetaxel+carboplatin; DP: cisplatin and docetaxel; EP: cisplatin+etoposide; GC: gemcitabine+carboplatin; IP: =cisplatin+irinotecan; LCC: large cell carcinoma; LN: lymph nodes; NA: data not available/not presented; NETs: neuroendocrine tumors; NP: vinorelbine-cisplatin; NS: never-smoker; NSCLC-NOS: NSCLC-not otherwise specified; OS: overall survivingtimeafter diagnosisoflungcancer; PD: progressive disease; PR: partial response; PD: progressive disease; RE: right eye; RT: radiation therapy; SCLC: small cell lung carcinoma; SD: stable disease; SqCC: squamous cell carcinoma; Surg: surgery; TC: carboplatin+taxol; TP: paclitaxel+cisplatin; *Metastases to adrenals, brain, bone, liver, distant lymph nodes, and abdominal organs specifically mentioned.									

单眼受累者中，右眼占35.7%（*n*=15），左眼占38.1%（*n*=16），双眼受累者占26.2%（*n*=11）。转移部位方面，脉络膜转移者占66.7%（*n*=28），为最常见受累部位；其他受累部位还有睫状体（*n*=1）、虹膜（*n*=4）、视网膜（*n*=1）、眼球（*n*=1）、视神经（*n*=3）、视盘（*n*=1）、眼眶（*n*=3）、眼睑（*n*=1）和结膜（*n*=1），有的患者同时存在多处部位受累。

肺癌临床特征方面，SCLC占21.4%（*n*=9），腺癌占47.6%（*n*=20），其他类型肺癌还包括神经内分泌肿瘤（*n*=5）、大细胞癌（*n*=2）、肺鳞癌（*n*=3）、肺腺鳞癌（*n*=1），2例NSCLC具体病理类别不详。我们发现眼转移患者常同时存在其他部位转移，其中脑转移者10例，占23.8%；骨转移者12例，占28.5%；肝转移者8例，占19.0%；肾上腺转移者6例，占14.3%；远处淋巴结转移者6例，占14.3%，腹腔转移者3例，占7.1%。

治疗方面，42例患者中，除外治疗不明者1例和仅对症治疗者2例，共39患者接受治疗，有25例采取了全身治疗联合眼部局部治疗，还有8例仅采取全身治疗，其中有6例仅采取了眼部局部治疗。在接受全身治疗的33例患者中，其中使用以铂类为基础的化疗者24例；单用伊立替康者1例；使用以铂类为基础的化疗联合贝伐珠单抗静脉注射者4例，其中1例为化疗进展后加用贝伐珠单抗治疗；靶向药物治疗者共5例，包括使用克唑替尼者2例，使用厄洛替尼者1例，2例患者一线化疗失败后改用厄洛替尼或吉非替尼治疗；外科治疗者2例。单用化疗25例患者，PR 3例，SD 4例，疾病进展（progressive disease，PD）者11例，未提及疗效者6例，总有效率12%，疾病控制率28%。化疗联合贝伐珠单抗静脉注射者，3例PR，1例SD。靶向治疗的5例均为3例PR，1例SD，1例PD。眼部局部治疗31例患者中，眼内注射贝伐珠单抗者13例，其中10例（76.9%）眼部症状得到控制，外放射治疗（external beam radiotherapy, ERBT）者4例，其他方式放疗者（具体不详）12例，16例接受放疗患者中12例（75%）患者眼部症状得到控制，手术切除者6例，其中4例患者接受了两种治疗。有42例患者中30例有生存信息，进行分析，患者中位生存时间13.00个月（95%CI: 7.80-18.20），但仅接受化疗16例患者中位生存时间为10.00个月（95%CI: 8.52-11.48）。

## 3 讨论

肺癌眼部转移是肺癌罕见转移的一种，其发病率低，但临床症状重，发生眼部转移后对于患者生活质量造成严重影响，预后差。本文回顾和总结9例肺癌合并眼部转移患者的临床特征和诊治结果，并回顾文献相关眼部转移病例，总结其治疗和预后，以期指导临床。

肺癌眼部转移是由于肿瘤血源性播散所导致。对于眼葡萄膜转移进行分析^[8]^，来自194例患者的229只眼睛，88%肿瘤位于脉络膜，虹膜转移（10%）和睫状体转移（2%）也有发生，18%出现双侧转移。脉络膜由眼球后部多个粗大的睫状后短动脉供血，且脉络膜血管之间有广泛的吻合支交通，血流丰富，易发生转移，因此，临床上最常发生眼部脉络膜转移。此外，眼眶转移、眼睑转移、结膜转移^[6]^、视网膜转移^[7]^、视神经转移^[37]^等也均有案例报道。本研究中的我院病例亦主要为脉络膜转移，仅有一例虹膜转移，而无其他部位的转移。文献回顾总结的42例患者，最常见的转移部位为脉络膜（66.7%），其他，例如虹膜、视神经、眼眶转移等亦有报道。

眼部转移导致的眼部的症状^[8]^包括视物模糊，疼痛，飞蚊症，视野缺损，肿块，红眼，闪光感，复视等，部分患者无症状。虹膜转移^[44]^也可出现表现瞳孔变形和继发青光眼。眼部转移病灶可以是多灶性也可以是单灶，单眼受累或双眼受累均可出现。然而，值得注意的是，在既往的文献报道中，有部分患者无眼部症状，进行常规筛查的过程中也可发现脉络膜转移^[3]^。对于我中心报道的9例患者，8例患者有不同程度的视力下降，也有患者出现飞蚊症、视野缺损、继发青光眼等等临床表现。此外，我中心虽然仅有9例肺癌患者有眼部症状，诊断为眼部转移，但是不能除外有更多的潜在患者，未进行筛查，然而，此类无症状患者是否需要常规进行筛查，目前仍然有争议，因为晚期肺癌的治疗仍以全身治疗为主，如无明确局部症状，无需进行局部治疗，因此，即使检测发现可疑转移灶，亦无需进行进一步治疗，因此是否进行筛查，尚需进一步探讨。

眼部转移瘤的诊断需结合临床检测及相应的影像学检测进行明确。对于以眼部症状为肺癌首发表现的患者，首诊科室为眼科。患者接受眼底检查、眼科超声、眼部核磁以及FFA，有经验的眼科医生可以直接诊断眼部转移瘤^[45]^，并进行进一步的全身肿瘤筛查发现肺癌。脉络膜转移癌的眼底病变检查具有一定的临床特征，转移瘤眼底病变通常为黄白色、宽基底、实性隆起，边缘不光滑，部分伴有渗出性视网膜脱离，FFA病灶呈斑驳样高低荧光混杂，对于转移癌有一定的提示意义，需要进一步筛查全身病变。必要时可依赖细针穿刺活检明确诊断^[46]^，极少数患者因治疗需要行眼部手术，病理明确肺癌眼部转移癌诊断。对于确诊肺癌患者，如果新发的眼部症状，积极进行相关检查，眼部超声和眼部核磁共振检查可以直观地显示眼部占位情况，结合眼底检查，易于诊断肺癌眼部转移。

对于肺癌合并眼部转移患者，治疗包括系统性抗肿瘤治疗和眼部局部治疗两方面，而患者的生存期取决于全身治疗疗效。化疗或靶向治疗对于眼部症状缓解和病灶控制也发挥一定程度的作用。然而，通过分析25例接受化疗的患者的临床疗效，发现即使接受了系统性化疗，合并眼部转移患者的全身疾病控制率仅为28%，治疗疗效极差，而联合应用静脉注射贝伐珠单抗及化疗4例患者^[9, 14, 22, 35]^，肿瘤均得到控制，提示对于此类患者，可尝试应用化疗联合抗血管生成治疗，但是病例数少，仍需进一步研究证实。对于有EGFR突变的患者，给予靶向治疗可以使得全身病灶及眼部转移灶均得到缓解^[36, 41]^。此外，伴有ALK重排^[39]^或是ROS-1重排^[11]^的患者，进行针对性的靶向治疗，也可以使得患者全身症状及眼部病灶得到控制。因此，对于合并眼部转移的NSCLC患者，积极进行驱动基因检测，并给予针对性靶向治疗。

对于眼部局部治疗是姑息性的，治疗的目的是维持视功能，改善生活质量。针对眼部转移的局部治疗方法仍然有限^[8]^。而体外放疗（远距离放疗），可抑制局部肿瘤生长，但可能导致相应的并发症，包括白内障，角膜炎，虹膜新生血管生成，放射性视网膜病变等。敷贴放射治疗（plaque radiotherapy）是一种近距离放疗方法，可以直接对脉络膜进行放疗，抑制肿瘤生长，主要的缺点是需要外科操作进行置入和取出敷贴。光动力疗法也是一个重要的方法，可以改善视力，仅有少量的视网膜出血，避免长时间的放疗和住院。局部应用抗血管生成治疗对于眼部转移病灶是一个可选的治疗方式^[9, 14]^，由于肿瘤转移的过程中，肿瘤新生血管起到重要的作用，因此玻璃体内注射贝伐珠单抗，可能起到局部抗肿瘤作用，改善视觉障碍，而且对于脉络膜转移导致的渗出性视网膜脱离，有一定的疗效。对于痛性盲眼，眼球摘除也是一种方法。对于症状轻的患者，在全身系统性抗肿瘤的基础上，进行观察，也是一种治疗选择。经局部治疗后，眼部病变可以66%变小，12%稳定，14%增大，3%复发，5%出现新转移，59%可以视力改善或维持原状，1年死亡率54%^[8]^。本研究中，经过眼部局部治疗，亦有较高的症状缓解率。

肺癌合并脉络膜转移相对较差，目前文献报道的诊断脉络膜转移后的中位生存时间仅为6个月-13个月^[8, 47]^。本文回顾文献的42例患者中，有30例有生存信息，进行分析，患者中位生存时间13.00个月（95%CI: 7.80-18.20），其中化疗患者中位生存时间仅10.00个月（95%CI: 8.52-11.48），与文献报道相一致。

对于出现视力模糊、眼部疼痛等眼部症状的肺癌患者，应警惕眼部转移，可完善眼部检查，进行排查。肺癌合并眼部转移以腺癌多见，脉络膜转移是最常见的眼部转移部位。眼部局部治疗（球内注射贝伐珠单抗、放疗、手术治疗）可控制局部症状，系统性化疗疗效差，化疗联合贝伐珠单抗或靶向治疗对于全身抗肿瘤治疗可能有效。
